# Gouty Tophi in Developed Countries: Uncovering Underlying Brain Diseases

**DOI:** 10.3390/diagnostics15192424

**Published:** 2025-09-23

**Authors:** Koji Hayashi, Mamiko Sato, Yuka Nakaya, Maho Hayashi, Toyoaki Miura, Hidetaka Matsuda, Yasutaka Kobayashi

**Affiliations:** 1 Department of Rehabilitation Medicine, Fukui General Hospital, 55-16-1 Egami-cho, Fukui 910-8561, Fukui, Japan; 2 Graduate School of Health Science, Fukui Health Science University, 55-13-1 Egami-cho, Fukui 910-3190, Fukui, Japan; 3 Department of Internal Medicine, Fukui General Hospital, 55-16-1 Egami-cho, Fukui 910-8561, Fukui, Japan; 4 Department of Gastroenterology, Fukui General Hospital, 55-16-1 Egami-cho, Fukui 910-8561, Fukui, Japan

**Keywords:** tophus, gout, gouty tophi, hyperuricemia, multiple intracranial hemorrhages, intracerebral hemorrhage, cerebrovascular disorders, dementia, developed countries

## Abstract

A 56-year-old man, accompanied by city hall staff, visited our neurorehabilitation clinic. Despite hyperuricemia being diagnosed several years ago, he refused treatment. He had no history of hypertension and antihypertensive drug use. He developed painful joint tophi around the age of 51, which were managed with over-the-counter painkillers. At age 54, a knee tophus was removed, histologically confirming gouty tophi. Subsequently, he lost his chef’s job, and his lifestyle deteriorated. Gouty tophi were observed in the right ear, knuckles, elbows, and ankles, with some ulceration. Blood tests showed anemia and hyperuricemia (10.1 mg/dL: reference 3.6–7.0 mg/dL). Chest–abdominal CT demonstrated calcification of the aorta. Brain MRI revealed an old putaminal hemorrhage and numerous microbleeds. Dementia (Clinical Dementia Rating: 1) was diagnosed based on neuropsychological testing. Public services and social assistance were arranged for him. This case is hypothesis-generating. In settings with adequate healthcare access, the presentation of severe, uncontrolled gouty tophi with poor engagement should prompt a selective, stepwise evaluation—beginning with cognitive screening and proceeding to neurologic assessment if indicated; routine preventive brain imaging is not recommended. The presence of lobar and deep microbleeds should be interpreted within the context of standardized diagnostic criteria and lesion distribution patterns to inform differential diagnosis.

**Figure 1:**
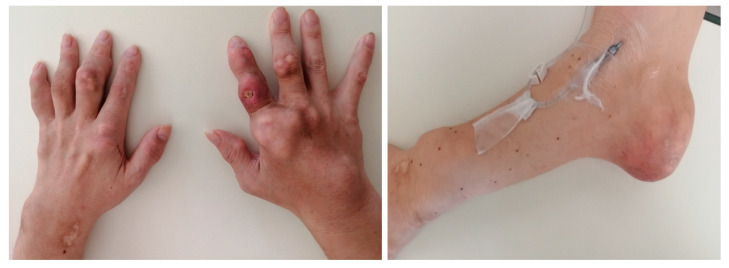
Photographs of Both Hands and the Left Elbow. Multiple nodules are observed on the fingers of both hands and the left elbow. Some of these nodules have ulcerated. Similar nodules are noted in various joints throughout the body. As the condition involves multiple arthritides with swollen joints in the limbs, the differential diagnosis of gouty tophi includes other rheumatic conditions such as rheumatoid nodules and rheumatoid arthritis; other forms of crystal arthritis include pseudogout; infectious diseases, including tuberculosis and infective endocarditis; and neoplastic diseases such as bone tumors and soft tissue tumors [1]. In our case, when the knee tophi was removed at age 54, monosodium urate (MSU) crystal analysis—which is considered the gold standard for diagnosing gouty tophi—was performed, leading to the diagnosis of gouty tophi. Gouty tophi are considered the characteristic feature of advanced or chronic gout with hyperuricemia as an underlying condition [2]. They represent an organized chronic foreign body granulomatous inflammatory response to monosodium urate crystals [2]. Tophi usually develop years after the initial gout attack in long-standing hyperuricemia [2]. A serious complication is ulceration, most common in the feet, especially the ankles and MTP joints [2]. Although gout incidence over recent decades is increasing all over the world, including North America, Europe, and East Asia [3], gouty tophi can be controlled with proper treatment, especially long-term urate-lowering therapies (ULTs) [2]. The exact incidence of gouty tophi remains unknown; however, in developed countries with good medical access, ULTs are widely available, making cases with multiple joint tophi, like this one, increasingly rare. Gouty tophi may not be benign lesions; they are associated with significant morbidity that severely impacts patients’ lives. The presence of tophi is strongly correlated with decreased health-related quality of life, affecting both physical and mental well-being [2,4]. Patients with tophi report significant functional limitations, including pain, restricted joint motion, joint deformity, and impairments in daily activities, leisure, and work productivity [4]. For instance, tophi in the foot and ankle, common sites of deposition, are associated with significant muscle force deficits, which can further impair mobility and function [5]. Complications such as ulceration, as seen in our patient, can lead to infection, delayed wound healing, and a further decline in quality of life while increasing healthcare costs [2]. The deterioration of our patient’s lifestyle and his loss of employment are consistent with the profound functional and psychological impact reported in individuals with severe tophaceous disease. Beyond the debilitating functional impact, the clinical significance of tophi extends to a direct association with mortality. The presence of subcutaneous tophi is a powerful and independent predictor of death. Gout is frequently accompanied by a cluster of comorbidities that are also major risk factors for atherosclerosis, such as hypertension, obesity, type 2 diabetes, chronic kidney disease, and dyslipidemia [4]. Indeed, studies have shown that a significant proportion of gout patients also suffer from these conditions, with one study reporting that 63% of gout patients had hypertension, 26% had type 2 diabetes, and 5% had chronic kidney disease [4]. Furthermore, and crucially, research directly links gouty tophi to increased mortality. A prospective study of individuals with recent-onset gout (<10 years) demonstrated that the presence of tophi at baseline was associated with an almost three-fold increased risk of all-cause mortality (Hazard Ratio 2.85, 95% CI 1.49–5.44) [6]. This elevated risk was independent of other factors such as age and renal function, and it applied to both cardiovascular and non-cardiovascular causes of death [6]. This evidence frames our patient’s severe clinical presentation not merely as a sign of chronic disease, but as a marker of a significantly heightened risk of death, underscoring the urgency to understand and address the barriers to his treatment. Although this patient did not have hypertension or diabetes, calcification of the aorta was confirmed, suggesting that his untreated gout had progressed to atherosclerosis.

**Figure 2:**
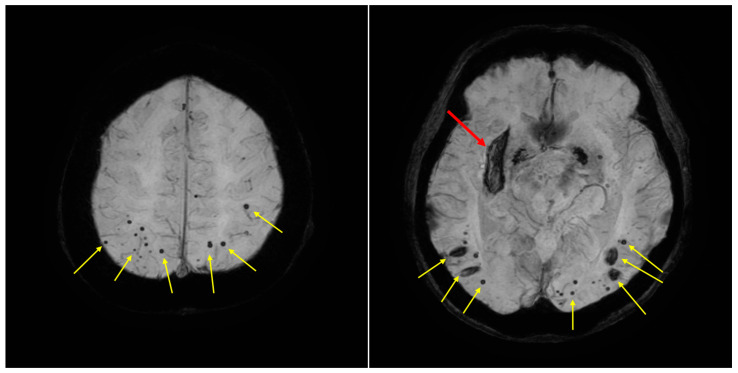
Brain Magnetic Resonance Imaging (MRI) Results. Susceptibility-weighted imaging (SWI) reveals multiple hypointense areas, including deep structures such as the right putamen (red arrow) and also distributed throughout lobar regions of the brain (yellow arrows). Given the presence of these multiple hemorrhages, both hypertensive small-vessel disease (HTN-SVD) and cerebral amyloid angiopathy (CAA) were considered in the differential diagnosis. However, the patient had no history of hypertension, and indeed, no instances of elevated blood pressure were noted during his hospitalization. This clinical information argued against a primary diagnosis of HTN-SVD. Attribution of these findings to either HTN-SVD or CAA should be made following standardized diagnostic criteria and careful evaluation of the lesion distribution patterns, which can distinguish between predominantly deep versus mixed lobar and deep microhemorrhages. Additionally, neuropsychological testing diagnosed him with dementia (Clinical Dementia Rating: 1). His cognitive decline and higher brain dysfunction may have contributed to his refusal to seek hospital care, resulting in the worsening of his gouty tophi. Hyperuricemia was associated with a significantly increased the risk of both stroke incidence (pooled RR, 1.42; 95% CI, 1.31–1.53) and stroke mortality (pooled RR, 1.53; 95% CI, 1.18–1.99) in meta-analyses [7]. For the risk of stroke mortality, the association between HUA and ischemic stroke (pooled RR, 1.39; 95% CI, 1.31–1.47) was more significant than that of hemorrhagic stroke (pooled RR, 1.13; 95% CI, 1.02–1.26) [4]. Additionally, hyperuricemia might be related to the progression of cerebral small vessel disease [8]. Consequently, in select cases with this severe phenotype—especially when accompanied by poor self-management—a stepwise approach beginning with cognitive screening and followed by neurologic evaluation as needed may help uncover the true barrier to effective treatment. These observations require confirmation in additional cases or a case series before broader recommendations can be made.

## Data Availability

The data presented in this study are available on request from the corresponding author. Due to patient privacy and ethical considerations, the data are not publicly accessible.

## Abbreviations

The following abbreviations are used in this manuscript:

MRI	Magnetic resonance imaging
MSU	Monosodium urate
SWI	Susceptibility-weighted imaging
CAA	Cerebral amyloid angiopathy
